# Learning curves in radiological reporting of whole-body MRI in plasma cell disease: a retrospective study

**DOI:** 10.1007/s11547-021-01391-3

**Published:** 2021-07-26

**Authors:** Davide Negroni, Alessia Cassarà, Alessandra Trisoglio, Eleonora Soligo, Sara Berardo, Alessandro Carriero, Alessandro Stecco

**Affiliations:** 1 AOU Maggiore Hospital, Via 2 Giugno 12, Galliate, 28066 Novara, Italy; 2 Nuovo Ospedale Degli Infermi Hospital, Biella, Italy; 3 San Paolo Hospital, Savona, Italy

**Keywords:** Whole-body MRI, Learning curves, Durie–Salmon PLUS, Plasma cell disease, Inter-observer agreement

## Abstract

**Background:**

The plasma cell disease is been studying by the whole-body MRI technology. However, the time requested to learn this radiological technique is unknown.

**Purpose:**

To esteem, quantitatively and qualitatively, the essential time to learn the whole-body MRI diffusion-weighted imaging with background body signal suppression in patients with plasma cell disease.

**Materials and methods:**

Between January 2015 and February 2017, three readers in-training with different levels of experience examined the anonymised and randomised whole-body MRI images of 52 patients with a diagnosis of plasma cell disease and analysed their morphological (T1w, T2w with and without fat suppression) and functional sequences. Reports of an expert radiologist were considered the standard of reference. Images were analysed in two sessions, during which each reader was timed. Readers reported the number of segments with lesions and staged the disease using the Durie–Salmon PLUS staging system. Weighted Cohen’s *ĸ* and *Z*-test were used to compare the trainees’ reports with those of the expert radiologist, and learning curves were drawn up to show changes between the two sessions.

**Results:**

Weighted Cohen’s *ĸ* of number of lesioned segments increased from 0.536 ± 0.123 to 0.831 ± 0.129 (Prob > *Z* under 0.005), thus approaching the goal of *ĸ* > 0.8. Trainees reached the level of experienced radiologist in terms of time by the 33rd patient. Agreement concerning the Durie–Salmon PLUS increased from 0.536 ± 0.123 to 0.831 ± 0.129 (Prob > *Z* under 0.005).

**Conclusions:**

The findings of this study demonstrate that whole-body MRI with DWIBS can be learned in about 80 reports and leads to a high level of inter-observer concordance when using the Durie–Salmon PLUS staging system.

## Introduction

Plasma cell disorders are a type of blood cancer in which plasma cells become malignant and can cause damage to the bones, kidneys, heart, bone marrow and immune system, and as a result can make patients sick. Monoclonal gammopathy of undetermined significance (MGUS), multiple myeloma (MM), plasmocytoma, lymphoplasmacytic lymphoma/Waldenström macroglobulinemia (LPL/WM), amyloidosis, and POEMS syndrome (Polyneuropathy, Organomegaly, Endocrinopathy, Monoclonal protein, and Skin changes) are included in this disorder [1].

Particularly, the MM has an annual incidence of 4–5/100,000 people per year, making it the most frequent primary malignant neoplasm of the skeletal system. It is a natural development of an MGUS, which has an annual rate of transformation into MM of 0.5–1%, and smouldering myeloma (SM), whose annual transformation rate is 10% during the first five years [2]. An early diagnosis is clinically significant as it greatly improves the patients’ long-term prognosis [3]. Adult MM mainly affects the axial skeleton, where most haemopoietic bone marrow is located [4]. Approximately 5% of all cases of plasma cell disorders are plasmacytoma, and the incidence is about 0.15 cases/100,000 person-years accounting for approximately 450 new cases per year in the USA [5].

Magnetic resonance imaging (MRI) is playing an increasing role in the diagnosis of MM because it is as sensitive as computed tomography (CT) in detecting plasma cell infiltration [6–9], and more sensitive than standard radiography [10–13] especially in patients who are asymptomatic [14]. Furthermore, whole-body MRI (WB-MRI) allows an even more detailed assessment of the degree and pattern of bone marrow infiltration, as well as the extramedullary extent of the tumour, and can potentially be used in the initial staging of patients with MGUS or MM (particularly in the case of extra-axial lesions) [15] and when selecting early therapeutic strategies [10]. The 2014 diagnostic criteria of the International Myeloma Working Group identify WB-MRI as the most sensitive means of detecting skeletal and extra-skeletal MM invasion [2], and its use can be expected to increase in the near future because its high level of contrast gives it advantages over CT, especially when imaging soft-tissue [16].

However, the multi-planar, multi-parametric, and multi-district capacity of WB-MRI, which is more closely related to nuclear medicine imaging than to typical radiology, may be a disadvantage during training because it requires transforming the radiological interpretation process (TRIP): thousands of images have to be managed and read [16, 17], and morphological and functional sequences reveal various disease patterns that need to be recognized in order to identify the different presentations of MM. Unfortunately, little is known about inter-observer agreement or the WB-MRI learning curves that visually represent improvements in inter-observer agreement during resident training. The only studies published so far relate to musculoskeletal and urological imaging [18, 19], while only one abstract with preliminary data about this argument was presented during the ESMRMB Annual Scientific Meeting [20].

The aim of this study was to make a qualitative and quantitative evaluation of the inter-observer agreement and learning curves of three residents being trained to use WB-MRI for the diagnosis of patients with plasma cell disorders.

## Materials and methods

### Patients

The trainees and teacher participating in this retrospective study reviewed the consecutive MRI scans of 62 patients affected by plasma cells disease, recorded between January 2015 and February 2017. The patient inclusion criteria were the availability of WB-MRI scans in our picture archiving and communication system (PACS); the absence of any other neoplasia (two patients excluded); the absence of MM treatment (eight patients excluded); and informed consent to undergo MRI.

All patients enrolled were divided into two equal groups, Group 1 and Group 2. This division was only a temporal grouping and was carried out in order to better describe the learning curve.

Being a retrospective study, the Ethical Committee approval was not required. Nevertheless, every patient signed an informed consent allowing the use of their clinical records anonymously for study purposes.

### Evaluation of imaging data

The three trainees who retrospectively examined the images for the first time between January 2015 and February 2017 were a senior resident with five-year experience in radiology (A.C.), a junior resident with two-year experience in radiology (A.T.), and a young resident with one-year experience in radiology (D.N.). They were introduced to the fundamentals of WB-MRI and the approach to MM by means of images from a previously archived teaching file presented during a short 30-min frontal lesson given by AS, an expert radiologist with more than 22-year experience in radiology and 10-year experience in WB-MRI, whose reports were used as the standard of reference for the study images.

The skeleton was divided into 15 regions (cranium, cervical column, thoracic column, lumbar column, ribs, sternum, right and left clavicle, right and left humerus, pelvis, right and left femur, and right and left scapula), which were analysed using functional and morphological MRI. Distal regions (tibia, fibula, tibial tarsal articulation, radius, ulna, and radio-carpal articulation) were excluded because of artefact effects. Every region was evaluated, and a record was made of the participants’ assessments of the number of lesions, the presence/absence of diffuse infiltration, and their answers to the dichotomous question, “Is this a lesioned segment? “. Each image was anonymised and analysed by each reader separately over a maximum of seven days with no more than three days between each seven-day period. The trainees were blinded to each other’s findings but, at the end of the analysis, they could read the official medical reports. The young resident clocked twenty of the expert radiologist’s usual reading sessions; the duration of the reading of the MRI sequences by each trainer during the first (Group 1) and second reporting session (Group 2) was also recorded. All of the participants (the expert radiologist and the three residents) evaluated the stage of the disease of each patient using the Durie–Salmon PLUS staging system.

### Lesion detection

In accordance with the state of the art, focal myeloma lesions were identified on the area of low signal intensity in T1-weighted spin-echo images and the area of high signal intensity in STIR images of the corresponding bone marrow, which indicate the focal accumulation of myeloma cells.

Widespread infiltration was diagnosed when the bone marrow signal was diffusely reduced in T1-weighted spin-echo images and increased in STIR images. Contrast media was not used [21]. Because of their high cellularity, focal MM bone marrow lesions can cause diffusion restriction and show enhanced signal intensity on DWI. The signal restriction in DWI was related to the presence of a morphological alteration in the T1 and T2 weighted sequences. In case of agreement, the area was considered as a lesion. The minimum size of the lesions was account as 5 mm. The segment number with this kind of lesion was recorded and underwent statistical analysis.

### MRI protocol

MR examinations were performed on a 1.5 T scanner (Achieva D-Stream; Philips Healthcare-Germany). We used the Q-Body coil for the analogical scanner and the Head and Neck and two Large Surface Body coils for the digital scanner to cover the entire body. Turbo Spin Echo T1w and T2w with and without fat suppression were performed in the sagittal plane; GE T1w and STIR sequences were performed in the coronal plane. Diffusion-weighted whole-body imaging with background body signal suppression (DWIBS) was performed in axial plane using the following parameters repeated for 5 stacks: TR/TE/TI = 7298/80/66.58 ms; FOV = 49 × 35 × 22 cm (RL, AP, FC); Matrix = 224 × 224; slice thickness = 5 mm; gap = 0; *b* value = 50,400,800 s/mm2; number of acquisitions = 3; EPI factor = 35; SENSE factor = 2. The average time of imaging acquisition between 45 and 50 min, depending on the patient’s compliance and habitus. As a result, all images made in the same sequence were merged during post-processing creating MIP and MPR images using Moby-view software.

### Statistical analysis

On the Durie–Salmon PLUS stage attributed to all of the patients in each group by each of the trainees, we identified those that each reader considered as requiring treatment, and then compared the results with the reference reports of the expert radiologist to identify the mean number of staging mismatches and any over-staging, an agreement concerning the need for treatment, and inter-observer agreement. The values were assessed using Weighted Cohen’s *k* and test *Z*.

**Fig. 1:**
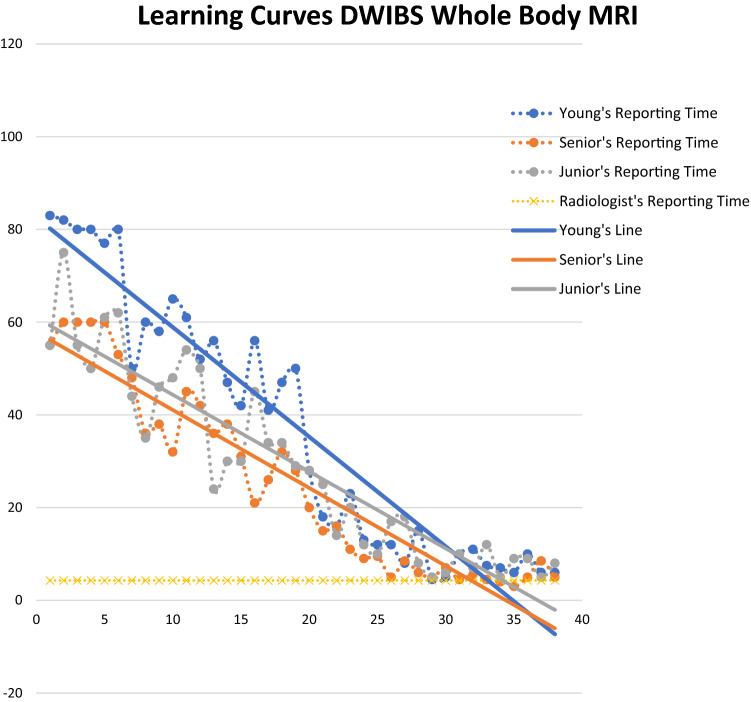
WB-MRI with DWIBS learning curve. The linear regression of the different curves is represented by the solid line identified as “line”. The intersection between this line and the "radiologist's reporting time" represents one of the goals of the study. *Senior* senior resident with five-year experience, *Junior* junior resident with two-year experience, *Young* Young resident without experience

The dichotomous question “Is this a lesioned segment?” was used to determine the total number of positive segments identified by each reader and the degree of inter-observer agreement for each subgroup, which was assessed using Cohen’s *ĸ* [22, 23]. Figure 1 is used to extrapolate the most descriptive straight-line equations. The angular coefficient (m) and *R*-squared were calculated because they, respectively, indicate the straight-line slopes and virtual line/time variability. Patients with a negative medical report were excluded from the learning curves to make the data homogenous. Both groups were statistically analysed using *t*-test, Fisher’s exact test and chi-square test to demonstrate the simple size comparability on demographic data, diagnosis, number of lesions and staging (alpha error = 0.05). All analyses were performed by using STATA version 13.1 (StataCorp. 2013. Stata Statistical Software: Release 13. College Station, TX: StataCorp LP).

## Results

Among 62 patients, 52 were included in the present study (38 with MM, eight with SM, and 6 with plasmacytoma; mean age 64 ± 13 years, range 37–86), divided into equal 2 groups (Group 1 = 26 patients and Group 2 = 26 patients) and each one was read by the three trainees. The two patient groups did not show any significant differences (*p* > 0.05).

### Inter-observer agreement

Figure 1 shows that the inter-observer agreement was almost perfect (*ĸ* ≥ 0.8).

#### First session

Using Durie–Salmon PLUS staging, the expert radiologist put 13 (50%) of the 26 patients in stage 1A, five (19%) in stage 1B, six (23%) in stage 2A, none in stage 2B, and two (8%) in stage 3A; the senior resident put 11 (42%) in stage 1A, seven (27%) in stage 1B, six (23%) in stage 2A, none in stage 2B, and two (8%) in stage 3A; the junior resident put 13 (50%) in stage 1A, six (23%) in stage 1B, five (19%) in stage 2A, none in stage 2B, and two (8%) in stage 3A; the young resident put 10 (38%) in stage 1A, eight (31%) in stage 1B, six (23%) in stage 2A, one (4%) in stage 2B, and one (4%) in stage 3A. The weighted Cohen’s *ĸ* analysis showed that inter-observer agreement between the trainees and the experienced radiologist was *ĸ* = 0.590 ± 0,124 for the senior resident, *ĸ* = 0.583 ± 0.125 for the junior resident and *ĸ* = 0.435 ± 0.121 for the young resident; the mean *ĸ* value of all three readers was 0.536 ± 0.123 (Table 1).

**Table 1:** Durie–Salmon PLUS staging system: Cohen *ĸ* values and SD (standard deviation)

		Cohen's *ĸ*	SD
RAD	G1	0.691	0.194
SEN	G2	0.837	0.196
RAD	G1	0.769	0.196
JUN	G2	0.831	0.193
RAD	G1	0.546	0.187
YOU	G2	1	0.196
Mean	G1	0.669	0.192
	G2	0.889	0.195

*RAD* radiologist with 22-year experience, *SEN* senior resident with five-year experience, *JUN* junior resident with two-year experience, *YOU*  young resident

Comparison of the assessments of the trainees with those of the experienced radiologist showed that the mean number of mismatches was 7.666 ± 1.699 (Table 2), and Cohen’s *ĸ* analysis showed that inter-observer agreement was an average of 0.717 ± 0.095 (range 0.691 ± 0.123–0.735 ± 0.153). The need-for-therapy agreement was 19/20 (95%) for all three readers. The disease-presence *ĸ* value was 0.691 ± 0.194 for the senior resident, 0.7692 ± 0.196 for the junior resident and 0.546 ± 0.187 for the young resident; the mean *ĸ* value of all three readers was 0.669 ± 0.192.

**Table 2:** Durie–Salmon PLUS staging system: study patient staging

	Durie–Salmon PLUS staging results												
*G1*													
RAD	1A	1A	1A	1B	2A	2A	1A	1A	2A	1A	1B	1A	1B
SEN	1B	1A	1B	1B	1A	3A	1A	1A	2A	1A	1B	1A	2A
JUN	1A	1A	1A	1B	1A	3A	1A	1A	2A	1A	1B	1A	2A
YOU	1B	1A	1A	1B	2A	2A	1A	1A	1B	1A	1A	1A	2A
RAD	1A	2A	1A	2A	1A	3A	2A	1A	3A	1B	1A	1A	1B
SEN	1A	2A	1B	2A	1A	2A	2A	1A	3A	1B	1A	1A	1B
JUN	1A	1B	1B	2A	1A	2A	2A	1A	3A	1B	1A	1A	1B
YOU	1B	1B	1B	2A	1A	2A	2A	1A	3A	2B	1B	1A	1B
*G2*													
RAD	1A	1A	3A	1A	1B	1B	1B	2A	1A	2A	1A	1B	1A
SEN	1A	1A	3A	1A	1B	1B	1B	2A	1A	2A	1A	1B	1A
JUN	1A	1A	2A	1B	1B	1B	1B	1B	1A	2A	1B	1B	1B
YOU	1A	1A	2A	1B	1B	1B	1B	1B	1A	1B	1A	1B	1A
RSD	1A	2A	1B	1A	1A	1A	2A	2A	3A	1B	2A	1A	1A
SEN	1A	2A	1B	1B	1A	1A	2A	2A	3A	1B	2A	1A	1A
JUN	1A	2A	1B	1B	1A	1A	2A	2A	3A	1B	1B	1A	1A
YOU	1A	2A	1B	1B	1A	1A	2A	2A	3A	1B	2A	1A	1A

*G1* first session group, *G2* second session group, *STR* radiologist with 22-year experience, *SEN* senior resident with five-year experience, *JUN* junior resident with two-year experience, *YOU* young resident

#### Second session

Using Durie–Salmon PLUS staging, the radiologist with 22-year experience put 12 (46%) of the 26 patients in stage 1A, six (23%) in stage 1B, six (23%9 in stage 2A, none in stage 2B, and two (8%) in stage 3A; the senior resident put 11 (42%) in stage 1A, seven (27%) in stage 1B, six (23%) in stage 2A, none in stage 2B, and two (8%) in stage 3A; the junior resident put eight (31%) in stage 1A, 12 (46%) in stage 1B, five (19%) in stage 2A, none in stage 2B, and one (4%) in stage 3A; and the young resident put 10 (38%) in stage 1A, 10 (38%) in stage 1B, five (19%) in stage 2A (19%), none in stage 2B, and one (4%) in stage 3A. The weighted Cohen’s *ĸ* analysis showed that the inter-observer agreement between the trainees and the experienced radiologist was *ĸ* = 0.725 ± 0.122 for the senior resident, *ĸ* = 0.769 ± 0.132 for the junior resident and *ĸ* = 1.000 ± 0.133 for the young resident; the mean *ĸ* value of all three readers was 0.831 ± 0.129.

Comparison of the assessments of the trainees with those of the experienced radiologist showed that the mean number of mismatches was 4.000 ± 1.633 (20% of the analysed patients). Cohen’s *ĸ* analysis showed that inter-observer agreement was an average of 0.816 ± 0.100 (range 0.787 ± 0.200–0.843 ± 0.000). The mean number of over-staged patients was 2.000 ± 0.817 (the equivalent of 11.5% of the analysed patients): two for the senior resident, three for the junior resident, and one for the young resident. The need-for-therapy agreement increased to 20/20 for all three trainees. The disease-presence *ĸ* value was 0.837 ± 0.196 for the senior resident, 0.831 ± 0.193 for the junior resident and 1.000 ± 0.196 for the first-year resident; the mean *ĸ* value of all three readers was 0.889 ± 0.195.

#### MRI

Of the 780 analysed segments, the expert radiologist and senior resident both reported that 104 were MRI-positive for multiple myeloma lesions; the junior resident reported 100 positive segments, and young resident reported 96 positive segments. The mean number of lesions was 4.423 ± 7.707 (range 0–33). The two residents and the young resident identified 77.000 ± 0.817 lesions (74%), missed 27 ± 0.817 (26%) and reported 17.333 ± 0.471 falsely negative lesions (16.7%). These percentages, respectively, changed from 72.4 to 75.6%; from 27.6 to 25%; and from 19.2 to 14.1% (Table 3).

**Table 3:** Inter-observer concordance: Cohen *ĸ* and standard deviation (SD) values

		Cohen's *ĸ*	SD
RAD	G1	0.59	0.124
SEN	G2	0.725	0.122
RAD	G1	0.583	0.125
JUN	G2	0.769	0.132
RAD	G1	0.435	0.121
YOU	G2	1	0.131
Mean	G1	0.536	0.123
	G2	0.831	0.129

*RAD* radiologist with 22-year experience, *SEN* senior resident with five-year experience, *JUN* junior resident with two-year experience, *YOU* young resident

### Learning curves

**Fig. 2:**
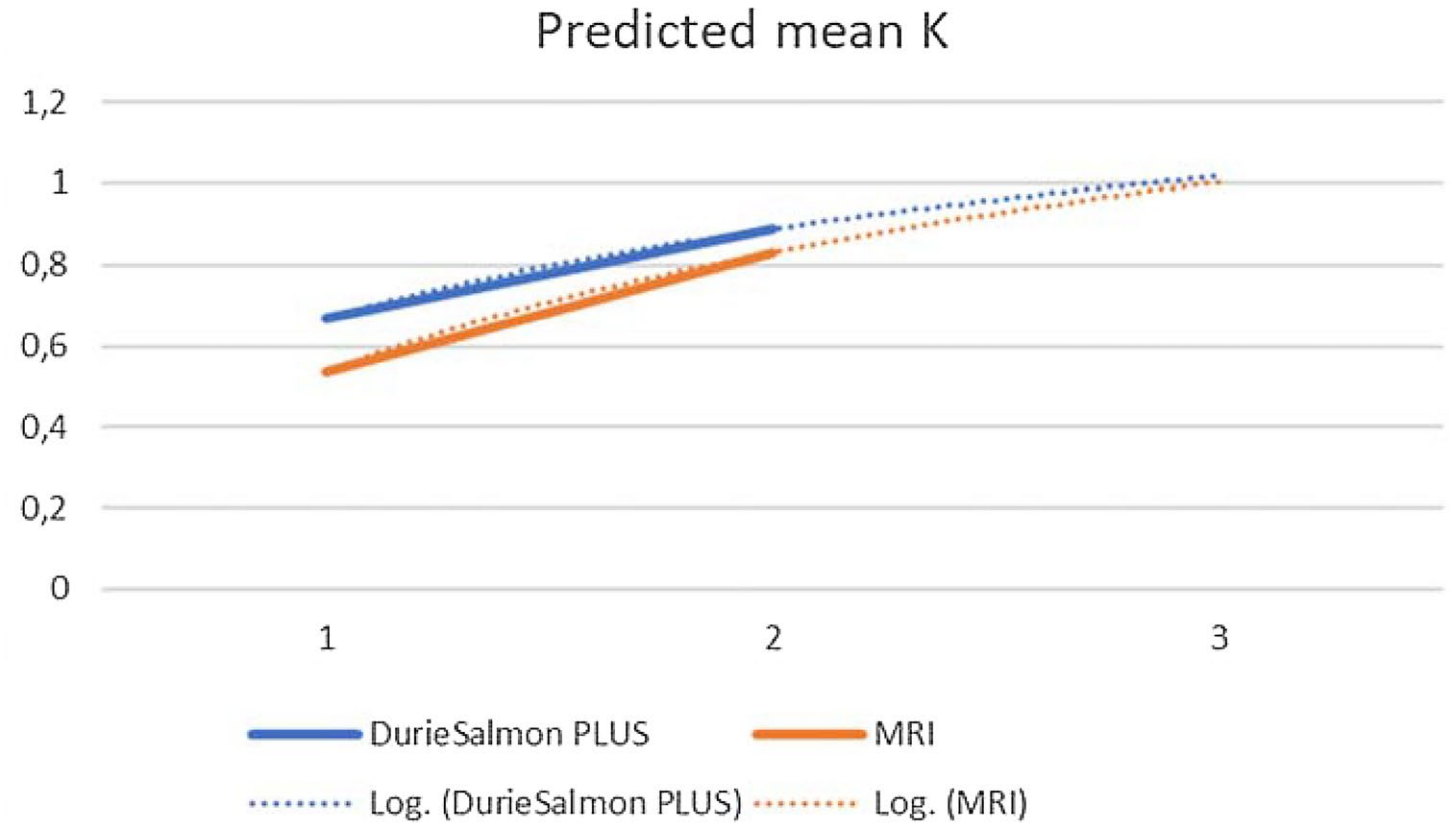
WB-MRI with DWIBS learning curve, logarithmic prediction. The two lines represent the average of the measurements obtained by the three trainees with several years of experience regarding the calculation of Durie–Salmon PLUS and the number of lesions on MRI

**Fig. 3:**
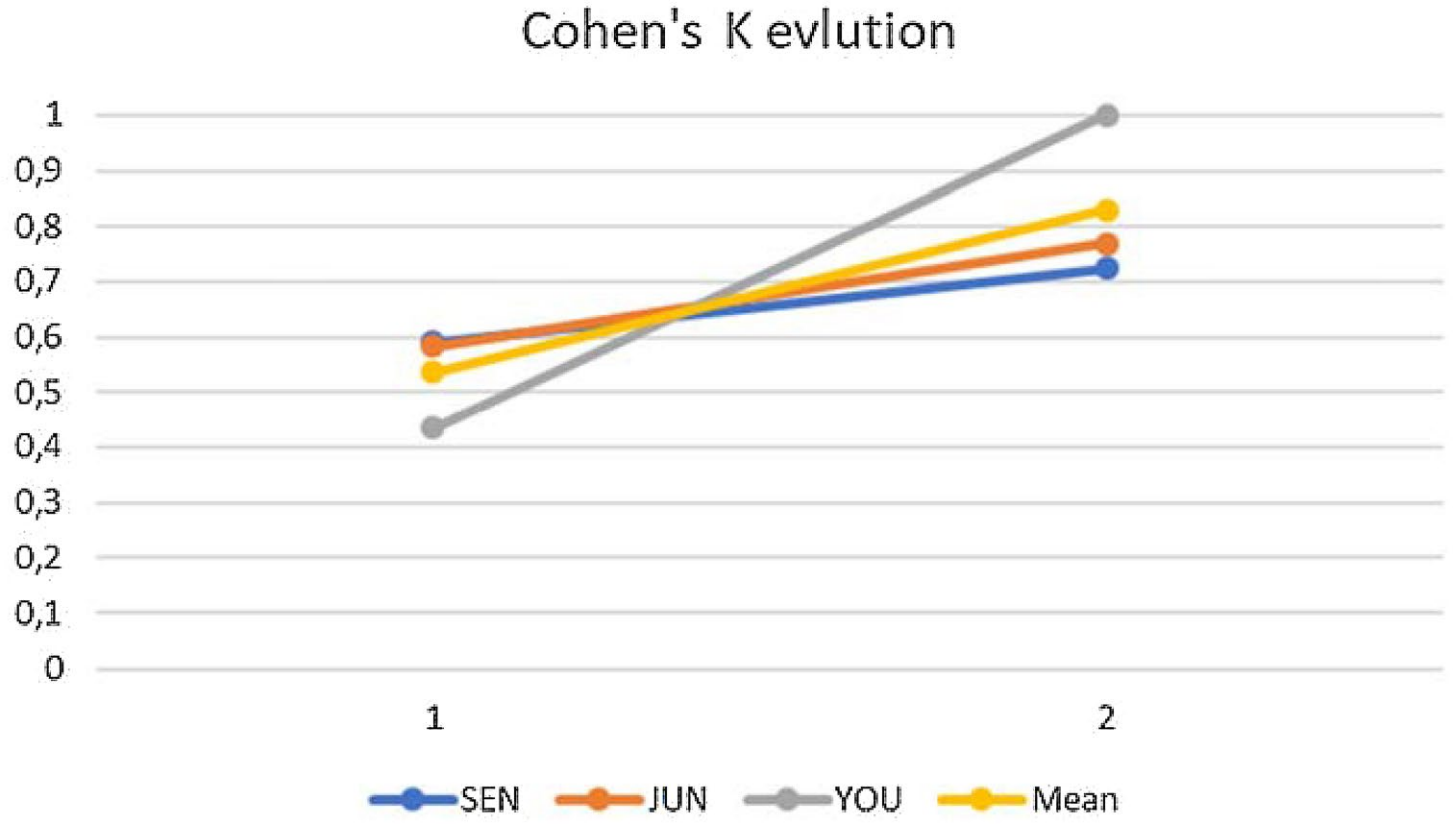
WB-MRI with DWIBS, Cohen’s k learning curve. This graph represents the evolution over time of the *ĸ* (*y* axis) between Group 1 and Group 2 (values on the *x* axis) of the trainees. *SEN* senior resident with five-year experience, *JUN*  junior resident with two-year experience, *YOU*  Young resident without experience

Figure 2 shows the logarithmic prediction of the curves. Figure 3 shows the behaviour of the Cohen *ĸ* values describing the inter-observer variations between the three trainees and the standard of reference.

#### First session

The angular coefficients (m) and R-squared values extrapolated from the DWIBS learning curves (Fig. 1 and Table 4) were, respectively, 2.07 ± 0.18 and 0.72 ± 0.12, with little variation from the linear regression line. The reading times of all the trainees decreased by 50%, while remaining approximately 27 min longer than the 4.5 min of the experienced radiologist (mean time after the 26th patient: 31.33 ± 8.74 min).

**Table 4:** First (G1) and second (G2) reporting session: straight-line *R*-squared and angular coefficient

		MRI G1	MRI G2
Senior	*m*	2.03	1.68
	*R*-square	0.8	0.89
Junior	*m*	1.92	1.66
	*R*-square	0.58	0.85
Young	*m*	2.27	2.36
	*R*-square	0.77	0.91
*m*-Value	*m*	2.07	1.9
	*R*-square	0.72	0.88
SD	*m*	0.178, 979	0.398, 497
	*R*-square	0.119, 304	0.030, 551

*Senior* senior resident with five-year experience, *Junior* junior resident with two-year experience, *Young* young resident without experience, *m*-value mean value, *SD* standard deviation

#### Second session

The MRI in this session included functional sequences (Fig. 1 and Table 4). The mean DWIBS “*m*” and *R*-squared values were, respectively, 0.723 ± 0.104 and 0.527 ± 0.104. The junior resident obtained the lowest R-squared value (0.377), which varied widely from the linear regression line. The senior resident touched the radiologist’s straight-line at eight points (crossing it at one), whereas the junior resident and the young resident touched it at two points.

Analysis of the first learning curve as a whole (Fig. 1) showed that the mean angular coefficient (m) and R-squared values were, respectively, 1.903 ± 0.330 and 0.890 ± 0.021 (Table 4).

At the 32nd patient, the senior resident spent as time as the expert radiologist’s one to report the MRI; for the junior resident this happened at the 34th patient and at the 33rd patient in the case of the young resident. The most descriptive equation of development was polynomial regression (polynomial mean *R*-squared = 0.918 ± 0.028 vs the straight-line mean *R*-squared = 0.877 ± 0.028). This was due to faster learning in the first session than in the second, but the difference was not enough to be described by logarithmic curves (log mean *R*-squared = 0.816 ± 0.027).

The intersection point between the regression straight-line and the expert radiologist’s line was approximately at the 32nd patient in the case of the senior resident, the 34th patient in the case of the junior resident, and the 33rd patient in the case of the young resident. The most descriptive equation of development was polynomial regression (polynomial mean *R*-squared = 0.918 ± 0.028 *vs* the straight-line mean *R*-squared = 0.877 ± 0.028). This was due to faster learning in the first session than in the second, but the difference was not enough to be described by logarithmic curves (log mean *R*-squared = 0.816 ± 0.027).

The *k* goal (*k* = 1) was esteem by logarithmic regression: the senior resident and the junior resident could reach the target in other 70–80 sessions. This esteem could not be performed to a young resident because he scored a *k* = 1.

## Discussion

MRI was employed for new and different dimensions, including the intramedullary and extramedullary cellular clusters in oncohematology. In this field, the diagnostic questions from clinical physicians are becoming more specific and the Radiology is getting a key role in the patient’s therapy. To deal with the advance, the radiologist physician should be highly trained. The study aimed was to determine the process of learning how to detect plasma cell diseases by using WB-MRI with DWIBS.

There are few papers about learning curves in the diagnostic radiology literature [18, 24], and those that have been published so far have concerned musculoskeletal MRI and sonography, radiotherapy [25], the prostate [19], and interventional radiology [26, 27]. The models presented in these studies report mathematical models of prediction through the use of logarithmic, exponential and sigmoid curves.

In particular, Tiago Rocha in 2017 reported the learning curves of facet joint injection under fluoroscopic guidance, through the use of mechanical simulators. By comparing the fluoroscopy time employed by the operator with the number of procedures performed, an inverse logarithmic curve was obtained [27].

Our study, using a similar mathematical model, obtained a high R coefficient (log mean *R*-squared = 0.816 ± 0.027). Although the polynomial regression model turns out to have a greater coefficient of R than the logarithmic one (polynomial mean *R*-squared = 0.918 ± 0.028 > log mean *R*-squared = 0.816 ± 0.027), the latter was chosen as the predictive study model, based on the results of the study by Dias et al. [27]. For the time taken by the trainees to reach the time taken by the experienced radiologist, it was necessary to report 33 MRIs of patients with plasma cell disease. These data were obtained by analysing the learning curves obtained and observing the intersection between the curves of the trainees and that of the expert radiologist (Fig. 1, solid lines).

The study of Mullaney of 2018, described learning in the ultrasound technique. The curves were obtained based on the evolution of Cohen's Kappa compared to an expert in the technique. As in our study, the Kappa was used to assess inter-rater agreement, to reach an ideal value of *ĸ* = 1 [24]. Although the absence of the timing used by the individual trainees does not allow a comparison of the respective curves, the conclusions are similar to our study: in about 80 reports, the residents could reach the experienced Radiologist, as our logarithmic prediction of the curves shows.

The logarithmic prediction, used to value the achievement of *ĸ* = 1 [24], is esteem with moderate reliability because it is based on the hypothesis that learning grows following a logarithmic pattern as shown in a few articles in the scientific literature [26, 27]. Using this mathematical model, it was possible to estimate the number of reporting sessions required (about 80).

This decrease in reading times and the concomitant increase in inter-observer concordance concerning positive segments and staging was an index of increased performance associated with the improvement in times. Training can be improved by a better understanding of learning processes and a rigorous assessment of training methods [18].

Concerning the MRI reporting methods, the residents had started to use WB-DWI as the first sequences, and DWIBS proved to be especially useful for detecting small lesions [25]. Although lymphadenopathy can be a confounding factor in patients with MM, it occurs in only 1% of patients. However, in our patients, occasional lymph nodes showed restricted diffusion due to their high cell density and the suppression of nearby adipose tissue [25], and this proved to be a source of error during the training.

It is also worth pointing out that DWIBS evaluations of areas close to the heart and diaphragm may be affected by signal loss and artefacts due to incoherent tissue motion. However, although rib sites are difficult to study using MRI, DWI is more sensitive than a skeletal survey when it comes to detecting rib lesions [26]. The DWI 3D reconstruction and the sagittal plane helped the residents to detect lesions in this anatomical region and in column. The sagittal plane was the most used by our residents in trainees to research lesions in the spine especially along with the spinous processes.

In the evaluation of all the Cohen’s *ĸ,* the increase in the mean value, the low SD, and the agreement concerning the need for therapy were excellent and encouraging results. In Durie–Salmon PLUS staging, the young resident had achieved the perfect agreement with the expert radiologist. Nevertheless, identification errors can be found analysing the difference between their reports. The young resident reported only part of lesions, down esteeming the disease presence inside bone segments. This error could not find inside the Durie–Salmon PLUS *ĸ* value: the wide range allowed the same patient staging despite the down esteeming error.

### Study limitations

This study has some limitations. It was a retrospective study of a small and heterogeneous patient population (52 patients with MM at various disease stages). Future studies defining repeatability for individual disease types at the same time points in their clinical course could provide additional meaningful information. The number of lesions affects the time spent on reading. It would be useful in the future to stratify patients based on the number of lesions using, for example, Durie–Salmon PLUS staging. Increasing the duration of the study such as in the case of a prospective study would identify when residents in-training reach the level of an experienced radiologist. This would allow more precise learning data. In this study, the inter-observer segment agreement was not assessed: the low ratio lesion/segment and the absence of exactly lesions number on some few official reports did not allow reliable data.

Implications for Patient Care:

- Whole-Body MRI is now an assessed imaging method for several clinical conditions, mainly in the oncologic field; reporting a big number of images raise the issue on the necessary skills demanded to correctly perform the radiologic report in this case, thus affecting patient’s care
- Over-staging remained a problem: 11.5% of the patients were over-staged and this could have led to significant public healthcare expenditure and could have had a negative impact on the patient’s quality of life
- Following up the findings of this study could identify when residents can be considered no longer “in training” and ready to report MRI findings, thus could improving patient management and the radiological diagnosis of plasma cell diseases.

In conclusion, the study demonstrated that WB-MRI with DWIBS can be learned with no substantial differences between residents. The findings of this study demonstrate that WB-MRI with DWIBS can be learned in about 80 reports and leads to a high level of inter-observer concordance when using the Durie–Salmon PLUS staging system.

## Data Availability

All data and material are guarded by the corresponding author and are available in case of require.
